# Regulation of Inflammasome by microRNAs in Triple-Negative Breast Cancer: New Opportunities for Therapy

**DOI:** 10.3390/ijms24043245

**Published:** 2023-02-07

**Authors:** Liliana-Roxana Balahura (Stămat), Sorina Dinescu, Marieta Costache

**Affiliations:** 1 Department of Biochemistry and Molecular Biology, University of Bucharest, 050095 Bucharest, Romania; 2 Research Institute of the University of Bucharest, 050663 Bucharest, Romania

**Keywords:** triple-negative breast cancer, inflammasome, pyroptosis, microRNAs, targeted therapy

## Abstract

During the past decade, researchers have investigated the molecular mechanisms of breast cancer initiation and progression, especially triple-negative breast cancer (TNBC), in order to identify specific biomarkers that could serve as feasible targets for innovative therapeutic strategies development. TNBC is characterized by a dynamic and aggressive nature, due to the absence of estrogen, progesterone and human epidermal growth factor 2 receptors. TNBC progression is associated with the dysregulation of nucleotide-binding oligomerization domain-like receptor and pyrin domain-containing protein 3 (NLRP3) inflammasome, followed by the release of pro-inflammatory cytokines and caspase-1 dependent cell death, termed pyroptosis. The heterogeneity of the breast tumor microenvironment triggers the interest of non-coding RNAs’ involvement in NLRP3 inflammasome assembly, TNBC progression and metastasis. Non-coding RNAs are paramount regulators of carcinogenesis and inflammasome pathways, which could help in the development of efficient treatments. This review aims to highlight the contribution of non-coding RNAs that support inflammasome activation and TNBC progression, pointing up their potential for clinical applications as biomarkers for diagnosis and therapy.

## 1. Introduction

The breast is a complex structure, which is organized in 15 to 25 lobes of different sizes that are connected with lactiferous ducts that terminate in the nipple, ductal formations and glandular tissue, all of these being surrounded by fibro-adipose tissue [1]. Specifically, the adult breast is a tissue highly variable in conformation, adiposity and volume, due to the different proportions between adipose, fibrous and glandular tissue, among which are also found blood and lymphatic vessels. The corresponding distribution of fat and collagenous components differs among women and is influenced by hormonal, physiologic and environmental factors [2].

Breast cancer (BC) is a dynamic, aggressive and heterogeneous disease that is the principle cause of death among women worldwide, but that also affects men [3]. This neoplasm has the potential to be determined by exposure to both genetic and non-genetic risk factors such as gender, age, menopause, nulliparity, obesity, alcohol abuse and exposure to hormones, radiation or therapy [1]. The early detection and diagnosis of BC is based on screening techniques (ultrasound, mammography, contrast-enhanced digital mammography, magnetic resonance imaging and positron emission tomography), microwave imaging techniques (microwave tomographic, radar-based microwave imaging and radiometry), biomarker-based techniques (radioimmunoassay, immunohistochemistry, enzyme-linked immunosorbent assay and fluoroimmunoassay) and breast tissue biopsies, which are used to differentiate between malign and benign tumors [4]. 

Following a diagnosis of BC, it is necessary to stage it according to the American Joint Committee on Cancer (AJCC) tumor, nodes, and metastasis (TNM) system, as TNM staging is used to define and stratify the size of the tumors (T), the status of regional lymph nodes (N), and distant metastasis (M) [5]. TNM staging is divided into four classes: (I) clinical staging, which includes information from clinical examination; (II) pathological staging, which includes the affected anatomical formations; (III) post-therapy staging, which includes clinical and pathologic information; and (IV) restaging, if necessary [6]. 

In order to establish the most efficient treatment for patients, the histological classification is completed via the molecular classification of BC that is based on the expression profiles of the estrogen receptor (ER), progesterone receptor (PR), human epidermal growth factor receptor 2 (HER2) and Ki67 antigen. The main molecular subtypes of breast cancer are luminal A (ER positive, PR positive and HER2 negative), luminal B (ER positive, PR negative and HER2 negative), HER2-enriched (ER negative, PR negative and HER2 positive), triple-negative breast cancer (TNBC), basal-like (ER negative, PR negative and HER2 negative), claudin low (ER negative, claudin negative, vimentin positive, E-cadherin low) and normal breast-like (adipose tissue gene signature) [7]. Luminal and HER2-enriched subtypes are associated with good prognoses and are highly responsive to therapy, resulting in a greatly improved outcome, while TNBC is the most aggressive subtype of BC, which is characterized by a high cell proliferation rate and a tendency to relapse [8]. Experimental studies indicate the presence of two main pathways involved in low-grade and high-grade breast tumorigenesis; low-grade BCs are regularly ER positive, PR positive and HER2 negative, while high-grade BCs are ER negative, PR negative and HER2 positive [9].

The mechanism of BC is dependent on the genetic modification and molecular processes that determine initiation, transformation and progression from normal tissue to tumor tissue [10]. More than 90% of diagnosed BCs are associated with the mutation of specific genes, such as breast cancer genes 1 and *2* (*BRCA1* and *BRCA2*), *TP53*, phosphatase and tensin homolog (*PTEN*), serine/threonine kinase 11 (*STK11*), ataxia-telangiectasia mutated (*ATM*), BRCA1 interacting protein 1 (*BRIP1*) or the partner and localizer of BRCA2 (*PALB2*), etc. [11].

In addition, BC aggressiveness is supported by chronic inflammation considering the up-regulation of pro-inflammatory cytokines, such as interleukin (IL)-1β, IL-6, IL-18 or tumor necrosis factor-α (TNF-α), growth factors or free radicals [12]. The secretion and release of pro-inflammatory cytokines are associated with inflammasome complex activation. Inflammasome is a cytoplasmic multiprotein complex, composed of a sensor (NOD-like receptor protein (NLRP)), an adaptor (an apoptosis-associated speck-like protein containing a caspase-activation and recruitment domain (CARD) (ASC)) and effector molecules (pro-caspase). The inflammasome complex is involved in numerous physiological and pathological mechanisms, including different types of cancer, although the activation of this complex can have both a positive or negative impact on carcinogenesis [13]. First of all, inflammasome activity stimulates epithelial mesenchymal transition (EMT), metastasis and angiogenesis, while inhibiting apoptosis and enhancing tumor development. On the other hand, the inflammasome pathway is also associated with immune reactions and the programmed death of tumor cells through pyroptosis [14].

The dynamic interaction between BC and inflammasome is regulated by a complex molecular network, including non-coding RNAs (ncRNAs). NcRNAs can be classified into short non-coding RNAs (sncRNAs) or long non-coding RNAs (lncRNAs), based on their number of nucleotides [15]. Among the most studied sncRNAs are microRNAs (miRNAs), which are single-stranded ribonucleic acid molecules involved in diverse cellular processes (cell survival, proliferation and adhesion, motility, cell death, inflammation, carcinogenesis, etc.). According to their implication in tumorigenic mechanisms and metastasis, miRNA molecules can be classified into oncogenic miRNAs and suppressor miRNAs [16]. Increasing evidence has indicated that many miRNAs are involved in the regulation of the inflammasome complex (miR-7, miR-9, miR-20a, miR-21, miR-23a, miR-30e, miR-33, miR-132, miR-133, miR-146, miR-155, miR-223, miR-296, miR-377, miR-711, etc.) and that these molecules represent a link between inflammasome activity and BC, especially TNBC [17]. Recently, researchers’ attention has been directed towards targeting these molecules as possible therapeutic strategies for the treatment of BC, due to their significance in post-transcriptional regulation [18].

Considering the importance of this topic and the ongoing expertise, this review aims to highlight the molecular mechanisms involved in BC correlated with the significance of the inflammasome pathway and miRNAs. Furthermore, another target is to accentuate the complex network and dual role of miRNAs (oncogenic and suppressor), in doing so emphasizing RNA-based techniques’ potential for clinical applications for diagnosis and therapy.

## 2. Molecular Characteristics of TNBC

TNBC is a highly invasive and aggressive type of BC, which is characterized by the absence of ER, PR and HER2 expression. TNBC represents almost 20% of all diagnosed BC, being associated with resistance to chemotherapy, a predisposition to metastasis, poor prognoses and reduced survival rates [19]. Over the years, researchers have studied the molecular signatures of TNBC using advanced techniques and, according to gene expression profiles, Lehmann et al. [20], Burstein et al. [21] and Jezequel et al. [22] have all proposed different classification of TNBC in order to investigate possible therapeutic targets to improve the outcomes of TNBC (Table 1). Moreover, according to Lehmann et al., TNBC can be subdivided into six groups: basal-like 1, basal-like 2, mesenchymal, mesenchymal stem-like, immunomodulatory, and luminal androgen receptor TNBC [20]. Burstein et al. proposed a TNBC classification divided into four subtypes: basal-like immune-activated, basal-like immune-suppressed, mesenchymal, and luminal androgen receptor TNBC [21], while Jezequel et al. divided triple-negative tumors into three clusters: C1 (22.4%), C2 (44.9%) and C3 (32.7%) [22]. Classifying and understanding the particularities of TNBC allow for the development of personalized medicine, because each subtype has different characteristics and responses to anti-tumor therapy [23].

The breast tumor microenvironment (TME) is associated with chronic inflammatory reactions, with inflammatory infiltrate being a prognostic marker of cancer. Pro-inflammatory molecules together with their specific receptors are involved in the development of TNBC due to their capability to promote cell differentiation and angiogenesis, to recruit immune cells and to influence the immune system [46]. Lymphocytes, macrophages and fibroblasts are the most common cell types of the TME and secrete different factors (such as cytokines, chemokines, enzymes, growth factors, etc.), which are associated with an aggressive malignancy and high risk of metastasis. Pro-inflammatory mediators (IL-6, CCL2, COX2, and TNF-α) are generally up-regulated in breast tumor stroma; IL-1, IL-6, IL-8, IL-11, IL-18 and IL-23 are among the most studied inflammatory factors involved in inflammation, TNBC, invasion and metastasis [47]. In close correlation with the development of inflammatory reactions and TNBC is the activation of the NLRP3 inflammasome complex, with IL-1β and IL-18 being the two major cytokines activated by NLRP3 inflammasome, which promote tumor cell proliferation and invasion [48]. 

Over the years, new signaling pathways within breast TME have been discovered, which have opened up new perspectives for the development of strategies that aim to block these signals that promote tumorigenesis, angiogenesis and metastasis. Specifically, conventional therapeutic strategies are the gold standard for neoadjuvant treatment represented by a combination between anthracyclines and taxanes, capecitabine and taxane and ixabepilone monotherapy (paclitaxel, 5-fluorouracil, doxorubicin, cisplatin, carboplatin, abraxane, bevacizumab, cyclophosphamide, ixabepilone, capecitabine, etc.), adjuvant treatment (anthracycline-based drugs), surgery (mastectomy and lumpectomy) and radiotherapy [49]. 

Despite the heterogeneity and aggressiveness of TNBC, the corresponding standard of care (SOC) requires neoadjuvant chemotherapy, followed by surgery and adjuvant chemotherapy (Table 2). There are several treatment schemes for early diagnosed TNBC that are based on anthracycline and taxane administration, but which cause numerous adverse effects (nausea, fatigue, gastrointestinal toxicity, myelosuppression, alopecia, hypothyroidism, hyperthyroidism, pneumonitis, skin reactions, adrenal insufficiency, peripheral neuropathy, neutropenia, pyrexia, anemia, thrombocytopenia, electrolyte abnormalities, infection, etc.) and do not lower the relapse rate [50].

Although neoadjuvant chemotherapy is the standard treatment approach for diagnosed TNBC, the therapeutic potential of other agents that interfere with various molecular mechanisms (such as angiogenesis, the immune response, the cell cycle, etc.) have been tested or are currently under testing and approval (Table 3). The optimal treatment scheme for TNBC is a great challenge due to the disease’s heterogeneity and increased risk of relapse, so the researchers have explored several promising anti-tumor agents (pembrolizumab, bevacizumab, olaparib, sacituzumab govitecan, etc.) [53]. Other therapeutic approaches are represented by platinum salts (carboplatin, cisplatin, etc.) immunotherapies that target the programmed cell death-1 (PD-1) receptor/PD-L1 pathway, immune-checkpoint inhibitors, poly-adenosine diphosphate ribose polymerase (PARP) inhibitors or AKT inhibitors [54].

The molecular characteristics of TNBC tumors (heterogeneity and the chemoresistance mechanism) raise difficulties in establishing an effective conventional therapeutic strategy; therefore advanced therapeutic strategies have been developed. There are two main types of advanced therapeutic treatments: passive transport (or enhanced permeability and retention (nanoparticles)) and active transport (miRNA and aptamers) [49].

## 3. Molecular Mechanisms of Inflammasome Activation during TNBC

**Figure 1:**
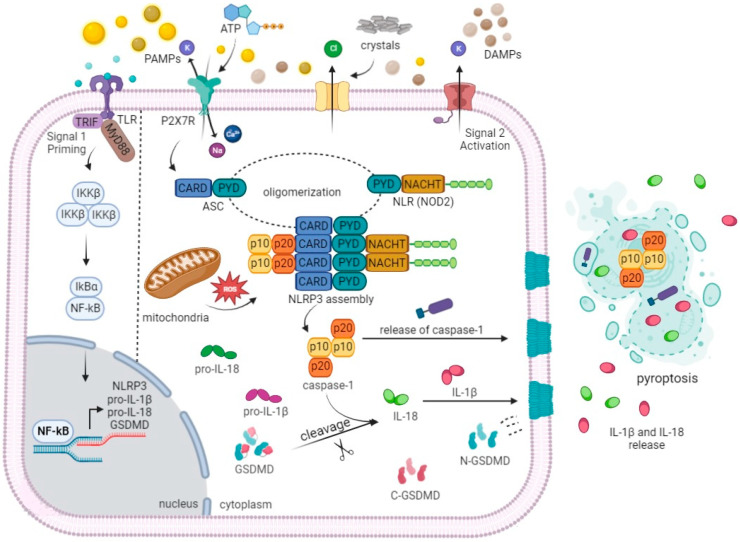
The mechanism of the NLRP3 inflammasome assembly. NLRP3 activation require two signals: priming and activation. The priming signal is induced by the binding of DAMPs or PAMPs and stimulates the up-regulation of inflammasome components through the NF-kB inflammatory signaling pathway. Together, these two signals determine the assembly of the NLRP3 inflammasome and the activation of CASP1, which supports the maturation and release of the pro-inflammatory cytokines (pro-IL-1β and pro-IL-18), and catalyzes the cleavage of GSDMD and pore formation into the membrane, leading to pyroptosis (created with BioRender.com).

Discovered in 2002 by Martinon et al., inflammasome represents the perfect structure bridging inflammation, tumorigenesis, angiogenesis and metastasis [45]. The inflammasome complex is a cytoplasmic multiproteic platform that comprises three components: the sensor molecule, the adaptor protein and the effector (Figure 1). The inflammasome pathway is activated by the recognition of specific stimuli, danger-associated molecular patterns (DAMPs), pathogen-associated molecular patterns (PAMPs), microbe-associated molecular patterns (MAMPs), and homeostasis-altering molecular processes (HAMPs) (such as ATP, toxins, K+ efflux, reactive oxygen species, etc.) by toll-like receptors (TLRs), the nucleotide-binding oligomerization domain (NOD) and leucine-rich repeat (LRR)-containing receptors (NLRs), the absent in melanoma-2 (AIM2)-like receptors (ALRs)), retinoic acid-inducible gene (RIG)-I-like receptors (RLRs) or C-type lectin receptors (CLRs). Inflammasome-related sensor molecules consist of pathogen recognition receptors (PRRs), such as TLRs, NLRs, ALRs, RLRs, CLRs and proteins that contain a tripartite motif, such as pyrin (also known as marenostrin or TRIM20) [45].

The activation of inflammasome is carried out by two main signals: priming and activation. The priming stimuli of the inflammasome are activate TLRs, the FAS-associated death domain protein and IL-1 receptor ligands, which activate the NF-kB signaling pathway, causing the up-regulation of inflammasome expression. In normal conditions, the expression of inflammasome components is relatively low and unable to initiate inflammasome activation. In this stage, two signaling molecules from the NF-kB pathway, MyD88 and TIR-domain-containing adapter-inducing interferon-β (TRIF), are involved in inflammasome regulation and pro-IL-1β secretion, but the expression of ASC, pro-caspase-1 and pro-IL-18 remains unmodified. Following the priming signal, the second signal, which is induced by the presence of DAMPs or PAMPs, is required in order to promote the ASC oligomerization, inflammasome assembly and caspase-1 (CASP1) activation [67].

These sensors can determine the oligomerization of the adapter protein ASC, also known as PYCARD, and the activation of the cysteine protease CASP1 [68]. After the detection of specific stimuli by the receptors, the ASC adapter proteins are assembled and form speck-like structures, which recruit pro-CASP1 that is cleaved into mature CASP1. Active CASP1 cleaves the pro-inflammatory cytokines (pro-IL-1β and pro-IL-18) and pore-forming gasdermin D (GSDMD) in order to promote the CASP1-dependent cell death, termed pyroptosis, with the pyroptosis’ mechanism being directed by the gasdermin family (GSDMA, GSDMB, GSDMC, GSDMD, GSDME and DFNB59) [69].

The classification of inflammasome is based on the structure of the receptor activating the signaling pathway, meaning that therefore this complex is divided into: canonical inflammasome-forming NLRs (NLRP1, NLRP2, NLRP3, NLRP6, NLRP7, NLRP9 and NOD-like receptor C4 (NLRC4) and NLRC5), non-canonical inflammasome-forming AIM2 (AIM2 and IFI16) and inflammasome-forming pyrin (MEFV) [70]. The canonical NLRP3 inflammasome is the most studied in the BC context, although several molecular aspects have yet to be investigated. Unlike canonical inflammasome, which involves CASP1 activity, non-canonical inflammasome requires the activation of human caspase-4/5 or murine caspase-11. Moreover, the study of non-canonical signaling is still ongoing and no details are yet known about the affected molecular mechanisms [71].

Breast tumor growth, progression and aggressiveness are promoted by NLRP3 inflammasome activation, pro-inflammatory cytokine secretion and the pyroptotic death of cancer cells. The overexpression of NLRP3, IL-1β, IL-18 and CASP1 is correlated with inflammatory signaling pathways, such as NF-kB, STAT1, STAT3, IL-1β or Wnt/β-catenin, developing an invasive TME, characterized by the release of reactive oxygen species (ROS), reactive nitrogen species (RNS), angiogenic factors, cytokines or chemokines [72].

Aggressive factors from the extracellular environment can cause alterations of cellular structures, leading to the release of pro-inflammatory mediators (cytokines, DAMPs or PAMPs, etc.). The presence of an inflammatory response in the TME is favorable for the initiation of programmed cell death (apoptosis, necroptosis, pyroptosis, etc.) [73]. 

Pyroptosis, or CASP1-dependent cell death, is characterized by cellular swelling, cell lysis, nuclear condensation and DNA fragmentation. Pyroptosis can have both pro-tumorigenic and anti-tumorigenic roles. The pro-tumorigenic role of pyroptosis is correlated with the NLRP3 and ASC oligomerization mechanism, while the anti-tumorigenic effects of pyroptosis are associated with the activation of the immunogenic signals and immune responses [74].

Following the activation of the NLRP3 inflammasome and the maturation of CASP1, the proteolytic cleavage of GSDMD into N-terminal and C-terminal domains is stimulated. The N-terminal domains of GSDMD attach to the cell membrane and form pores of 10–20 nm in diameter, with the GSDMD-derived pores allowing the influx of calcium or sodium ions and efflux of potassium, thus stimulating the inflammatory process [75].

Over the last years, there has been a special interest in the research field of the contribution of inflammasome complexes to the onset and progression of various diseases, such as obesity, diabetes or cancer. Advanced molecular studies have exposed the dynamic role of inflammasomes and the overlapping with the immune system and inflammatory-associated mechanisms. The connection between inflammasome complexes and the immune system is represented by the secretion and maturation of pro-inflammatory IL-1β, which activates the NF-kB signaling pathway and angiogenic signals, leading to tumor development [76].

The complex relationship among TNBC, inflammasome signaling, the immunosuppressive TME and resistance to chemotherapy requires meticulous investigation. Specifically, breast TME is characterized by the interaction between infiltrated immune cells, inflammatory cells, cytokines, chemokines and growth factors. According to Table 1, two of the criteria for the TNBC classification are represented by the involvement of the immune system and inflammation in the tumorigenic mechanisms, which implies personalized treatment strategies based on inflammatory and immune particularities [77]. In breast malignancies, an immunosuppressive TME is closely correlated with inflammasome signaling. Activation of immune mechanisms (cytokine and chemokine signaling pathways and T-cell receptor or B-cell receptor signaling, etc.) stimulates NOD-like receptor signaling leading to inflammasome activation. In addition, the activation of immune pathways (such as TLRs) stimulates the detection of DAMPs and PAMPs and induces intracellular signals that contribute to the recruitment of ASC adaptor protein and the oligomerization of inflammasome [78,79].

Over the years, clinical trials have revealed that the increased expression of NLRP3 components is associated with unfavorable prognoses and lower survival rates. The up-regulation of NLRP3 inflammasome in TNBC promotes cell migration and invasion, being associated with epithelial–mesenchymal transition (EMT) mechanisms and metastasis through CASP1-dependent mechanisms and the autocrine production of pro-inflammatory IL-1β [80].

Although the function of pyroptosis in the TME of TNBC has not yet been fully elucidated, studies have revealed that pyroptosis plays a dual role (both anti- and pro-tumorigenic). The activation of pyroptosis can thereby generate inflammatory reactions and immune responses that could enhance the sensitivity to immunotherapy [78]. Recent studies have further indicated that the activation of NLRP3 inflammasome stimulates the myeloid cells’ infiltration in breast TME [80]. On the other hand, pro-inflammatory cytokines are released into the TME and promote tumor growth. Additionally, the up-regulation of gasdermin B (GSDMB) is associated with poor prognoses and less sensitivity to HER2-targeted therapy [81].

Considering that inflammasome is implicated in TNBC development and progression, numerous studies have focused on discovering the potential anti-cancer strategies of inflammasome components, such as the identification of possible NLRP3, IL-1β or CASP1 inhibitors (MCC950 or AcYVAD-cmk) or the identification of ncRNA molecules that intervene at the transcriptional level. For example, anakinra, canakinumab or rilonacept are inhibitors of the IL-1 signaling pathway, which contribute to the initiation of apoptosis and blockade of tumor cells’ cycle progression [82]. As mentioned previously, inflammasome opens up new therapeutic perspectives for TNBC, which must be studied thoroughly.

## 4. Non-Coding RNAs in Oncology

The sequencing of the human genome indicated that approximately 98% of genes are non-functional or “junk DNA” while about 2% of them encode proteins. Moreover, technological advances in sequencing have revealed that the non-coding sequences are copied into RNA molecules that can assist fundamental biological functions, such as regulating gene expression, cell growth and organs’ development, or play an important role in the evolution of a wide variety of human diseases, including cancer, autoimmune disease, metabolic diseases or inflammation. Regarding the implication of ncRNAs in cancer, mutation or aberrations in the promoters of ncRNAs and the dysregulation of the enzymes involved in their biogenesis (Drosha, Dicer) can lead to altered gene expression levels, the normal expression levels of which are crucial for the normal functioning of the human body. Furthermore, up- or down-regulation of ncRNA expression is associated with cancer progression and metastasis [83].

**Figure 2:**
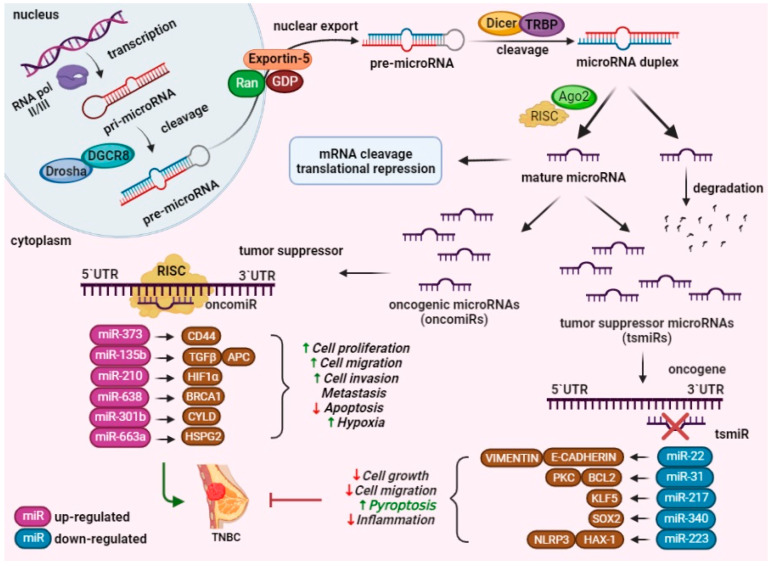
MiRNAs biogenesis and roles in TNBC progression. Genes are transcribed by RNA polymerase II/III into a pri-miRNA molecule, which is cleaved by DGRC8 and Drosha generating the pre-miRNA. The pre-miRNA molecule is exported into the cytoplasm via exportin-5, where Dicer and TRBP cleaves the pre-miRNA, resulting in the mature miRNA. The functional mature miRNA molecule is associated with AGO2 onto the RISC, leading to gene silencing via mRNA cleavage, translational repression and degradation. Additionally, the up-regulation of oncogenic miRNAs determines the suppression of target genes, while the down-regulation of tumor suppressor miRNAs stimulates the overexpression of target genes. MiRNAs assist essential biological processes such as cell growth, proliferation, migration and invasion, hypoxia, inflammation, apoptosis, pyroptosis and metastasis in TNBC (created with BioRender.com).

As mentioned previously, ncRNAs can be classified into sncRNAs or lncRNAs, based on their number of nucleotides [15]. Among the most studied sncRNAs are miRNAs, small interfering RNAs (siRNAs), small nucleolar RNAs (snoRNAs), transfer RNA (tRNA)-derived small RNAs (tsRNAs), and PIWI-interacting RNAs (piRNAs). MiRNAs are single-stranded ribonucleic acid molecules that incorporate between 19 and 25 nucleotides and regulate the expression of the target messenger RNAs (mRNAs). In the eukaryotic cells, a primary transcript (pri-miRNA) with multiple secondary structures is formed and is cleaved by two enzymes (Drosha and Dicer) in order to form a single hairpin structure precursor of microRNA (pre-miRNA) and miRNA duplex. Subsequently, these molecules are incorporated into the RNA-induced silencing complex (RISC) and are associated with Argonaute RISC catalytic component 2 (AGO2), leading to the cleavage of the complementary mRNA or suppression of translation (Figure 2) [15].

Among the most studied oncomiRs are miR-373, miR-135b, miR-210, miR-638, miR-301b, miR-663a, etc. (Figure 2). MiR-373 was first identified as a metastasis-promoting miRNA in breast cancer, being involved in tumor-cell proliferation, migration, invasion and metastasis via CD44 targeting [84]. The up-regulation of miR-373 determines the secretion and maturation of caspase-8, which is mainly responsible for the non-canonical activation of the inflammasome complex [85]. MiR-135b is an oncomiR that targets TGF-β in order to enhance cell proliferation and invasion [86], at the same time being involved in the inhibition of CASP1 expression and inflammatory reactions [87]. MiR-210 is also known to play a significant role in breast tumor biological functions, including tumor proliferation and metastasis via regulation of hypoxia and programmed cell death [88]. In the aberrant miR expression, miR-301 is up-regulated in TNBC and promotes tumor growth, cell proliferation, migration, invasion and tamoxifen resistance via the TLR4/NF-kB signaling pathway [89]. Moreover, recent studies have revealed that miR-663a is an inflammation-related miRNA that is frequently deregulated in TNBC, being involved in both apoptotic pathway and inflammasome activity through secretion and maturation of pro-inflammatory cytokines [90,91]. The findings of these studies discovered that miR-638 up-regulation might play a critical role in TNBC progression and inflammasome assembly via modulation of the ATP synthesis mechanism [92,93].

MiRNAs have been described as important regulators in tumor suppression, with miR-217 having been acknowledged as an inhibitor of tumor growth, inflammation and inflammasome, through its regulation of the inflammatory response and the induction of oxidative stress and programmed cell death [94]. Recent studies have indicated that miR-340 functions as a tumor suppressor and cell cycle regulator and that miR-340 could affect pyroptosis through NLRP3 inflammasome and the TLR4/NF-kB signaling pathway, subsequently leading to decreased inflammatory signaling [95,96]. MiR-31 is among the most frequently altered microRNAs in breast malignancies, and the altered expression of miR-31 is associated with the negative regulation of the NF-kB signaling pathway, inflammasome activation, the release of cytokines and cell death [97,98]. Additionally, miR-223 is an anti-inflammatory microRNA that acts as a tumor suppressor in TNBC. The published literature has demonstrated that miRNA-223 expression is associated with NLRP3 inflammasome inhibition and the inhibition of cell cycle progression [99,100].

The main anti-cancer strategy is to activate the programmed death of cancer cells. For example, pyroptosis is a caspase-and-gasdermins-mediated cell death, which can be regulated by drugs or genetic interventions. Recently, special interest has been given to the potential of pyroptosis to suppress TNBC through the recruitment of tumor-suppressed immune cells and the activation of anti-tumor immunity. Studies have indicated that CASP1-induced pyroptosis combined with immune checkpoint inhibitors (ICIs) can enhance anti-tumor immunity in vitro and in vivo [101]. In TNBC conditions, immune cells (CD8+ T cells, NK cells, etc.) stimulate the expression of gasdermins, then coordinate M1 macrophages and T lymphocytes infiltration, in order to induce pyroptosis to stimulate cell sensitivity and to activate tumor clearance [102]. In addition, the activation of pyroptotic pathway by anti-tumor drugs, such as paclitaxel and cisplatin, determines the inhibition of tumor proliferation and metastasis [103]. 

In the field of oncology, miRNA, tsRNA and piRNA are among the most studied sncRNAs, with numerous studies having focused on the investigation of the mechanisms of miRNAs regarding the molecular and cellular processes involved in cancer conditions. Their results have demonstrated that miRNAs are regulators that guide the expression of both coding and non-coding molecules, such as oncogenes (RAS, MYC, etc.) and tumor suppressors (TP53, BRCA1, etc.). More than that, miRNAs can function as oncogenes, inhibiting the tumor suppressor molecules’ activity, promoting accelerated cancer cell growth and finally encouraging the development of tumor formations. Tumors are subordinate to oncogenic miRNAs (oncomiR) expression for their survival and growth, due to the phenomenon of oncomiR addiction, making them promising potential therapeutic targets in anti-cancer treatment, thereby clearing the way to miRNA-based therapy [104,105]. On the other hand, miRNAs can also act either as tumor suppressors or oncogenes, depending on the progression of associated mechanisms [106]. Additionally, miRNAs can be used as molecular biomarkers because the aberrant expression of several miRNAs has been detected in multiple types of cancer. The expression level of a specific miRNA can be used to indicate the state of the cancer, to differentiate malignant tumors from benign ones and to indicate the type of cancer. Additionally, the molecular signatures of miRNAs outline the prognosis, anticipating the potential for metastasis and the response to therapy [107].

## 5. MicroRNAs Interplay with TNBC and NLRP3

MiRNA molecules are important regulators that target and interfere with mRNA translation, thus influencing gene expression. The aberrant expression of miRNAs disturbs normal biological mechanisms, such as cell growth, proliferation and migration, programmed cell death, and immune or inflammatory responses. The progression of BC (especially TNBC) is promoted by the abnormal expression of several miRNAs (Table 4). Involved in the development of TNBC are numerous up-regulated (miR-135b, miR-21, miR-301b, miR-221, miR-298, miR-449, miR-302b, miR-663a, miR-638, miR-137, miR-140, miR-155, miR-210, miR-9, miR-181, miR-373, etc.) and down-regulated miRNAs (miR-217, miR-211-5p, miR-185, miR-204-5p, miR-128, miR-340, miR-384, miR-200c, miR-31, miR-200a, miR-141, miR-27b-3p, miR-223, etc.) [108,109].

MiRNAs are the main modulators of gene expression, being involved in numerous normal and pathological mechanisms. Certain miRNAs are directly involved in inflammasome complex modulation into breast TME and also indirectly alter the inflammasome-specific markers by modulating other signaling pathways, such as mTOR, MAPK, cAMP/PKA, AMPK or IFN signaling pathways [146]. Additionally, recent studies have identified the potential of miRNAs to regulate pyroptosis of breast tumor cell through activation or suppression of various molecular networks [147]. Several research groups have carried out relevant studies that have contributed to the understanding of miRNA-mediated regulation of inflammasome assembly and pyroptosis activation in order to develop therapies that target the inflammasome [148].

Wang et al. investigated the influence of miR-200b on NLRP3, ASC, GSDMD, CASP1, IL-1β and IL-18 expression. Their results indicated that up-regulation of miR-200b determined the pyroptosis activation via regulation of the JAZF1/NF-kB signaling pathway. The tumor promoter JAZF zinc finger 1 (JAZF1) is a repressor of TGF-β-activated kinase 1 (TAK1), which is involved in NF-kB signaling cascade. The research group also observed that high expression of JAZF1 constrained the effect of miR-200b on the pyroptosis mechanism [149].

MiR-155-5p is another miRNA molecule that was found to be up-regulated in TNBC. Xu et al. evaluated the capacity of miR-155-5p antagomir to enhance the cytotoxic effects of cetuximab on tumor cells in vitro. The results demonstrated that the combination between miR-155-5p antagomir and cetuximab regulates GSMDE and CASP1 in order to stimulate the pyroptotic cell death and to decrease tumor growth. The in vivo experiments also confirmed that down-regulation of miR-155-5p increased the cytotoxicity of cetuximab, leading to pyroptosis and apoptosis activation in TNBC cells [124]. 

Bioinformatic analysis of miRNAs’ role in inflammasome-mediated breast tumorigenesis has revealed that certain miRNAs that are associated with the NLR family and consequently influence the prospect of TNBC. For example, NLRP3 is associated with miR-125, miR-146b, miR-200, miR-223-3p, miR-373, miR-520 or miR-548 [150,151], NLRP1 regulation is realized by miR-143-5p, miR-210 [152], and NLRP8 is modulated by miR-181 [153]. The modulation of inflammasome by these miRNAs has effects on TNBC through different signaling pathways, such as NF-κB, TGF-β or IL-6, which are also regulated by the same miRNAs (miR-146b, miR-520, miR-373, etc.) and in normal conditions inhibit TNBC cell growth and proliferation [154,155]. MiRNAs function as master modulators of inflammasome and pyroptosis-related TNBC by regulating the expression of multiple genes and mechanisms. Although some studies have revealed a piece of miRNA-associated networks, it is compulsory to expand the knowledge about inflammasomes and the molecular mechanisms of pyroptosis into the context of TNBC.

MiRNA molecules are involved in the regulation of NLRP3 components, leading to the overexpression of pro-inflammatory markers. MiR-223 is a non-coding molecule and NLRP3 regulator that can bind to the 3′UTR region of NLRP3 mRNA, inhibit the translation mechanism and alter the release and maturation of pro-inflammatory cytokines [156]. Among the numerous other microRNAs involved in NLRP3 regulation is miR-22, which acts as a tumor suppressor. The increased expression of miR-22 is correlated with NLRP3 assembly difficulties, decreased cell proliferation rate and metalloproteinase 2 (MMP2), MMP9, vimentin, and N-cadherin low-expression profiles [157]. The aggressive behavior of cancer is due to the miR-21 activity, which inhibits the NLRP3 assembly by regulating NLRP3 phosphorylation and lysine 63-ubiquitination and alters the normal functions of the innate immune system [158]. 

Among the microRNA molecules that regulate NLRP3 inflammasome is miR-9. As well, miR-9 has been reported to perform important functions in tumor initiation, inflammation and immune regulation [159]. MiR-9 can suppress the activation of the NLPR3 inflammasome and NF-kB signaling, thereby determining the inhibition of CASP1 expression and the secretion of pro-inflammatory factors (IL-1β and IL-18). The up-regulation of miR-9 is also associated with embryonic lethal abnormal vision-like protein 1 (ELAVL1) regulation, anti-inflammatory mechanisms and the inhibition of pyroptosis cell death [126].

MiRNAs have great potential to become effective therapeutic targets in TNBC treatment, considering the critical functions of microRNAs in TNBC progression and the modulation of NLRP3 components’ expression. Patients diagnosed with TNBC are still lacking an effective therapeutic response to chemotherapy, radiotherapy, surgery and immunotherapy. Therefore, it is necessary to develop new powerful and modern therapies that target the molecular mechanisms involved in the development of triple-negative breast tumors.

MiRNA molecules are decisive for oncogenic processes that have been intensively studied in recent years. For example, numerous studies have focused on the investigation of targeted therapies to silence (for oncogenes) or overexpress (for tumor suppressors) specific miRNAs [111,160]. However, miRNA-based therapeutic applications represent a subject that has not yet been fully elucidated, although these types of treatments have been evaluated in several pre-clinical studies, with the yielded results being deemed satisfactory. MiRNA-derived therapies are based on nucleotide hybridization, so the advantage of using them is that targeted sequences can be easily approached and that they can easily target combinations of RNAs. Although miRNA-based therapy indeed offers many advantages, the method for their delivery still remains a challenging subject [161,162,163].

MiRNA-based therapeutic strategies used in TNBC are either nucleic-acid-based strategies or drug-based strategies. Nucleic-acid-based treatments are divided into two groups: miRNA replacement (miRNA mimics) and anti-miRNA therapy, with the anti-miRNA treatment also being subdivided into two categories depending on the mechanism of action (miRNA antagonists or antagomiRs and miRNA sponges) [129].

MiRNA replacement or miRNA mimic is used in order to restore the defective tumor suppressor miRNA expression and to inhibit breast tumorigenesis progression. The delivery system of miRNA mimics is well received by normal cells [163]. For example, Li and her team explored the mechanism and role of miR-1290 in a radio-treated TNBC context, employing the transfection method for both miR-1290 mimic and miR-1290 inhibitor. Specifically, miR-1290 is a critical mediator that induces tumor proliferation and metastasis, being a potential biomarker for the diagnosis and prognosis of BC. The results of the first experiments carried out indicated that after radiotherapy the levels of NLRP3, ACS and CASP1 did not significantly differ between the radiosensitive and radioresistant MDA-MB-231 cells. Subsequently, the researchers evaluated the profile of miR-1290 in radiosensitive and radioresistant cells and concluded that the expression of miR-1290 was higher in the resistant MDA-MB-231 cells than in those that were sensitive. In the final part of their experiments, the transfection of the miR-1290 mimic and inhibitor demonstrated that the miR-1290 mimic had a positive impact on cell viability and inhibited NLRP3 inflammasome assembly via ASC and CASP1 suppression. Regarding pyroptosis, the miR-1290 inhibitor determined the up-regulation of CASP1, IL-1β and IL-18 by targeting NLRP3. One conclusion of this complex study was that miR-1290 promoted the radioresistance of MDA-MB-231 cells [163]. In addition to this study, which highlights the potential of miR-1290 as a key regulator of pyroptosis, other studies have revealed that many miRNAs are involved in positive regulation of pyroptosis through NLRP3 inflammasome activation, such as miR-21 [164] or miR-155-5p [124].

Anti-miRNA therapy is based on three main approaches: genetic knockout, anti-sense oligonucleotides (meaning antagomiRs) and miRNA sponges. AntagomiRs are modified anti-sense oligonucleotides that consist of ribose residues 2′-O-methylation, cholesterol residues 3′-conjugated and phosphorothioate linkages. AntagomiRs affect miRNA-related pathways by binding and inhibiting the activity of specific oncomiRs involved in TNBC development and progression [165], and the effectiveness of antagomiR therapy varies depending on tissue type and the method of delivery to the targeted tissue. This approach can be used as complementary sequences attached to the miRNAs or in the form of locked nucleic acid oligonucleotides, which contain a bond between the 2′-oxygen and 4′-carbon at each nucleotide, in order to inhibit TNBC progression and metastasis [166]. In 2016, Conde and their research team developed a RNA-triple-helix structure comprised of a miR-205 mimic and antagomiR-221 in order to evaluate a new platform for local anti-cancer therapy using both oncomiR inhibition and tumor suppressor miR replacement approaches. After miR delivery, the obtained data suggested that miR-205 and miR-221 targeted laminin subunit gamma 1 (LAMC1) protein and influenced TNBC by stimulating cell adhesion, proliferation, migration and metastasis, in both in vitro and in vivo conditions; on the other hand, miR-205 up-regulation was a consequence of an increased p53 profile. The concluding remarks of this study revealed that miR-221 and miR-205 inhibition affected E-cadherin, Slug and Snail expression, reducing TNBC cell migration and invasion. This new strategy also determined VEGF down-regulation, suppressing angiogenesis in TNBC contexts [167]. MiR-205 supports breast tumors’ growth and inhibits apoptosis due to its capacity to mediate the assembly mechanism of the NLRP3 complex. The activation of NLRP3 is possible due to the increase in NLRP3, ASC and CASP1 expression and the development of an inflammatory response under the control of miR-205 [168]. The up-regulation of miR-205 has also been reported to decrease steroid receptor co-activator (SRC) expression, which is involved in inflammation through the regulation of pro-inflammatory IL-18 expression [169,170]. On the other hand, miR-221 is a key modulator of breast tumorigenesis and inflammatory responses [171]. The up-regulation of miR-221 has anti-inflammatory effects, and suppresses oxidative stress and programmed cell death in TNBC, due to the decrease in Bcl-2 expression and the suppression of the NLRP3/ASC/CASP1 signaling pathway, as well as inflammatory reaction [114].

MiRNA sponges represent another anti-miRNA strategy, which contain multiple binding sites (between four and sixteen) complementary to the target miRNAs and which have the capacity to inhibit other miRNAs that contain between two and seven specific sequential nucleotides [172]. Advanced studies indicate that miRNA sponges can be transcribed by both RNA polymerase II and III promoters, because their transcripts are stable as a result of capped 5′ and 3′ polyadenylated tails [173]. Additionally, numerous endogenous miRNA sponges that influence the profiles of TNBC-specific biomarkers have been studied. For example, Zhang et al. designed a self-assembled DNA nanosponge embedded with doxorubicin, which mimics the role of miRNA sponge transcripts for the adsorption and clearance of miR-21. The obtained sponge-like delivery system determined an increased efficiency of anti-tumor therapy due to cell apoptosis-specific gene regulation [174]. More interestingly, the potential of such a miR-21 sponge-based strategy can also be used to prevent metastasis through the negative regulation of colony stimulating factor-1 (CSF-1), a biomarker for malignancy and metastasis [175].

On the other hand, drug-based strategies focus on the regulation of miRNAs through the activity of certain intercalating agents (stilbenes, curcumin, flavonoids, histone deacetylases inhibitors, etc.) that bind to miRNA [176]. The regulation of miRNAs by flavonoids has a negative impact on tumor cells’ proliferation, metastasis and EMT, but also modulates important mechanisms, such as angiogenesis, inflammation, inflammasome, immune responses, etc. [177]. The research activities carried out in this field have demonstrated that quercetin can influence the expression of certain oncomiRs, such as miR-21, miR-155 or let-7, leading to BC regression by programmed cell-death stimulation and increasing the sensitivity of tumor cells to chemotherapy [178]. In addition, miR-146a, miR-146b, miR-605 and miR-381 expression is modulated by quercetin, determining tumor growth inhibition and apoptosome assembly [179].

Over the years, the role of miRNA in TNBC progression has been intensively studied, with the results of these studies having highlighted the existence of numerous miRNAs involved in the modulation of the inflammasome complex, such as miR-223-3p, miR-7-5p, miR-22-3p, miR-33, miR-9, miR-155, etc. Their mechanism of action is based on binding 3′UTR of NLRP3 mRNA conserved regions and interfering with translation [180]. For example, Xie et al. studied the influence of miR-33 on NLRP3 inflammasome activation. The obtained results confirmed that miR-33 attenuates mitochondrial oxygen consumption and increases the production of cellular ROS, given that it is responsible for the secretion of IL-1β and the increased expression of NLRP3 and caspase-1 in macrophages [181]. On the contrary, Zhang et al. studied the possibility of inactivating the inflammasome and implicitly the growth of breast tumor cells targeting miR-233. Firstly, the researchers demonstrated the NLRP3 knockdown results in a decrease in breast tumor cell proliferation and migration, due to apoptosis initiation. Secondly, they used miR-233 mimics in order to suppress NLRP3 expression and NLRP3-dependent mechanisms, such as expression levels of ASC, IL-1β, IL-18 and CASP1. At the end of their study, the researchers stated that miR-233 activity inhibits the NLRP3 activation pathway, leading to the suppression of tumor cell growth, migration and invasion [18]. Another study, conducted by Hu et al. highlighted the role of miR-155 in the aggressiveness of TNBC due to the NLRP3 inflammatory pathway. Additionally, miR-155 interference decreased the MDA-MB-231 cell proliferation and ability to form colonies, stimulated the apoptosis of tumor cells and inhibited secretion of pro-inflammatory mediators [182].

## 6. Conclusions

In summary, this review integrates miRNAs and their functions into the inflammasome-dependent TNBC progression, as miRNAs are key regulators of gene expression and tumorigenesis, being involved in NLRP3 inflammasome assembly and pyroptosis cell death initiation. Due to the heterogeneity and aggressiveness of TNBC caused by genetic and environmental factors, the development and validation of effective targeted strategies has been challenging. Over the past few years, wider attention has been paid to the activation of NLRP3 in oncogenic pathways and to the targeted therapeutic approaches based on miRNAs combined with anti-tumor drugs, in order to increase the survival rate of patients. The results of the original experiments are encouraging, although the elucidation of the molecular manipulation of inflammasome via microRNAs in order to improve TNBC treatment is imperative.

## Figures and Tables

**Table 1:** Molecular comparison between three proposed classifications of TNBC.

TNBC Classification	Method of Analyses	Number of Patients	Subtypes	Abnormal Mechanisms	Relevant Markers	Therapeutic Strategies	Refs.
The Vanderbilt Subtype	K-means clustering	586	Basal-like 1	Cell cycle Cell proliferation DNA damage response	MYC, PIK3CA, CDK6, AKT2, KRAS, FGFR1, IGF1R, CCNE1, CDKN2A/B, BRCA2, PTEN, MDM2, RB1, TP53, KI67	PARP inhibitors HDAC/DNMT inhibitors Natural-killer therapy Cisplatin,	[20,24,25,26]
			Basal-like 2	EGFR, MET, NGF, Wnt/β-catenin, TP63, IGF1R signaling pathway Glycolysis Gluconeogenesis	TP53, TP63, EGFR, MET, BRCA1, RB1, PTEN, CDKN2A, UTX	mTOR inhibitors Growth factor inhibitors (lapatinib, gefitinib, cetuximab, etc.)	[20,24,27]
			Immunomodulatory	Th1/2, IL-7, IL-12 signaling pathway	TP53, CTNNA1, DDX18, HUWE1, NFKBIA, APC, BRAF, MAP K4, RB1, CTLA4, PDL1	PD1/PDL1/CTLA4 inhibitors Cisplatin PARP inhibitors	[20,24]
			Mesenchymal-like	Cell motility Cell proliferation Cell differentiation Wnt, TGFβ, Notch signaling pathway Epithelial-mesenchymal transition	PTEN, RB1, TP53, PIK3CA, VEGFR2, PI3KCA	mTOR inhibitors Drugs targeting epithelial–mesenchymal transition Abl/Src inhibitor Dasatinib	[20,24,28]
			Mesenchymal stem-like	Cell motility Cell differentiation Growth factor signaling Epithelial–mesenchymal transition Low proliferation	ABCA8, PROCR, ENG, ALDHA1, PER1, ABCB1, TERT2IP, BCL2, BMP2, THY, HOXA5, HOXA10, MEIS1, MEIS2, MEOX1, MEOX2, MSX1, BMP2, ENG, ITGAV, KDR, NGFR, NT5E, PDGFR, THY1, VCAM1, VEGFR2	mTOR/MEK/PI3K inhibitors, Src antagonists Antiangiogenic drugs Abl/Src inhibitor Dasatinib	[20,24]
			Luminal androgen receptor	Steroid synthesis, porphyrin metabolism, Androgen/estrogen metabolism	DHCR24, CD166, FASN, FKBP5, APOD, PIP, SPDEF, CLDN8	Anti-AR therapy PI3K/CDK4/6 inhibitors	[20,24,26,29]
The Baylor Subtype	Non-negative matrix factorization	198	Luminal androgen receptor	Steroid hormone biosynthesis Porphyrin and chlorophyll metabolism PPAR signaling pathway Androgen and estrogen metabolism Hormonale-mediated signaling	TP53, PI3KCA, AKT1, ERBB2, ERBB4, CDK4/6, AR, MUC1, ER, CDH1, KRT7, KRT8, KRT18, KRT19, XBP1, FOXA1	Anti-AR/MUC1 therapy	[21,30,31,32,33,34]
			Mesenchymal	Cell motility Epithelial–mesenchymal transition Focal adhesion TGF-β signaling pathway Adipocytokine signaling pathway	PIK3CA, PTEN, STAT3, IGF1, prostaglandin, TGF-β, Wnt, β-catenin, PDGFRα, c-Kit, ABC transporter	TKI/RAS/mTOR inhibitor Growth factor inhibitors	[21,30,31,32,33]
			Basal-like immunosuppressed	Mitotic cell cycle Mitotic prometaphase M phase of mitotic cell cycle DNA replication DNA repair Immune response Innate immune response	VTCN1, TP53, CENPF, BUB1, PRC1, VTCN1, MS4A6A, MTBP, FGFR2, BARD1, RNASE6	VTCN1 inhibition	[21,31]
			Basal-like immune-activated	Cytokine–cytokine receptor interaction T cell receptor signaling pathway B cell receptor signaling pathway Chemokine signaling pathway NF-kB signaling pathway	CCR2, CXCL13, CXCL11, CD1C, CXCL10, CCL5, STAT	Drugs targeting stat signal transduction molecules and cytokines	[21,31,35]
The French Subtype	Fuzzy clustering	194	Cluster 1	Luminal androgen receptor enriched	AR, Hsp90, PI3K, FGFR4, TTN, TNR, PKHD1L1, SPTA1, NCKAP5, COL15A1, ANKRD11, MYLK	Anti-AR therapy	[36,37,38]
			Cluster 2	Basal-like with low immune response High M2-like macrophages High pro-tumorigenic Low anti-tumor immune response	CCL2, CCL5, CCL18, CCL10, CXCL22, IL4, IL8, IL10, IL13, TGFβ1, CD206, CD204, VEGF, Aginase1, PIK3CA, NF1, AKT1, FBN3, ABCC1, DNHD1	M2 inhibition Repolarization of M2 into M1 macrophages	[20,38,39,40,41,42,43,44,45]
			Cluster 3	Basal-enriched High immune response Low M2-like macrophages Low pro-tumorigenic High anti-tumor immune response	IL-1β, IL-6, IL-12, IL-23,CXCL9, TNF-α, CCL2, IFNγ, GSF10, DNAH1, CDH23, AHNAK2, GTF3C1	Repolarization of M2 into M1 macrophages	[38,41,45]

Abbreviations: ATP-binding cassette (ABC), ATP-binding cassette subfamily a member 8 (ABCA8), ATP-binding cassette subfamily b member 1 (ABCB1), ATP-binding cassette subfamily c member 1 (ABCC1), tyrosine-protein kinase ABL1 (Abl), AHNAK nucleoprotein 2 (AHNAK2), protein kinase B (AKT), aldehyde dehydrogenase 1 family, member A1 (ALDHA1), ankyrin repeat domain 11 (ANKRD11), antigen-presenting cell (APC), apolipoprotein D (APOD), androgen receptor (AR), BRCA1-associated RING domain protein 1 (BARD1), B-cell lymphoma 2 (BCL2), bone morphogenetic protein 2 (BMP2), serine/threonine-protein kinase B-Raf (BRAF), breast cancer type susceptibility (BRCA), mitotic checkpoint serine/threonine-protein kinase (BUB1), chemokine (C-C motif) ligand (CCL), cyclin E1 (CCNE1), C-C chemokine receptor type 2 (CCR2), cluster of differentiation (CD), cadherin (CDH), cyclin-dependent kinases (CDK), cyclin-dependent kinase inhibitor (CDKN), centromere protein F (CENPF), claudin 8 (CLDN8), collagen type XV alpha 1 chain (COL15A1), cytotoxic T-lymphocyte associated protein 4 (CTLA4), catenin alpha 1 (CTNNA1), C-X-C motif chemokine ligand (CXCL), DEAD-box helicase 18 (DDX18), 24-dehydrocholesterol reductase (DHCR24), dynein axonemal heavy chain 1 (DNAH1), DNA methyltransferase 1 (DNMT), epidermal growth factor receptor (EGFR), endoglin (ENG), epidermal growth factor (ERBB), fatty acid synthase (FASN), fibrillin 3 (FBN3), fibroblast growth factor receptor (FGFR), FK506 binding protein 5 (FKBP5), forkhead box A1 (FOXA1), growth differentiation factor 10 (GSF10), general transcription factor IIIC subunit 1 (GTF3C1), histone deacetylase (HDAC), homeobox A cluster (HOXA), heat shock protein 90 (Hsp90), HECT, UBA and WWE domain containing 1, E3 ubiquitin protein ligase (HUWE1), interferon (IFN), insulin-like growth factor 1 (IGF1), insulin-like growth factor 1 receptor (IGF1R), interleukin (IL), integrin subunit alpha V (ITGAV), kinase insert domain receptor (KDR), marker of proliferation Ki-67 (KI67), Kirsten rat sarcoma virus (KRAS), keratin (KRT), mitogen-activated protein kinase 4 (MAPK4), mouse double minute 2 homolog (MDM2), Meis homeobox 1 (MEIS), mitogen-activated protein kinase kinase 1 (MEK), mesenchyme homeobox 1 (MEOX), tyrosine-protein kinase Met (MET), membrane spanning 4-domains A6A (MS4A6A), msh homeobox 1 (MSX1), MDM2 binding protein (MTBP), mechanistic target of rapamycin kinase (mTOR), mucin 1 (MUC1), myosin light chain kinase (MYLK), NCK associated protein 5 (NCKAP5), neurofibromatosis type 1 (NF1), nuclear factor kappa-light-chain-enhancer of activated B cells (NF-kB), NFKB inhibitor alpha (NFKBIA), nerve growth factor (NGF), nerve growth factor receptor (NGFR), ecto-5′-nucleotidase (NT5E), poly (ADP-ribose) polymerase (PARP), programmed cell death protein 1 (PD1), platelet-derived growth factor receptor (PDGFR), programmed death ligand 1 (PDL1), period circadian regulator 1 (PER1), phosphoinositide 3-kinase (PI3K), phosphatidylinositol-4,5-bisphosphate 3-kinase catalytic subunit alpha (PIK3CA), phosphatidylinositol phosphate (PIP), polycystic kidney and hepatic disease 1-like protein 1 (PKHD1L1), polycomb repressive complex 1 (PRC1), protein C receptor (PROCR), phosphatase and tensin homolog (PTEN), retinoblastoma susceptibility gene (RB1), ribonuclease a family member K6 (RNASE6), SAM pointed domain-containing Ets (SPDEF), spectrin alpha, erythrocytic 1 (SPTA1), steroid receptor coactivator (Src), signal transducer and activator of transcription (STAT), telomerase reverse transcriptase (TERT), transforming growth factor (TGF), tumor necrosis factor (TNF), tenascin-R (TNR), transient tachypnea of the newborn (TTN), ubiquitously transcribed tetratricopeptide repeat X chromosome (UTX), vascular cell adhesion molecule 1 (VCAM1), vascular endothelial growth factor (VEGF), vascular endothelial growth factor receptor 2 (VEGFR2), V-set domain-containing T-cell activation inhibitor 1 (VTCN1), wingless/integrated (Wnt), X-box binding protein 1 (XBP1).

**Table 2:** SOC for TNBC.

Approach	Class of Agents	Examples of Therapy	Mechanism of Action	Refs.
Neoadjuvant	Anthracycline + Taxane	Doxorubicin + Cyclophosphamide + Paclitaxel Epirubicin + Cyclophosphamide + Nab-paclitaxel	Inhibition of DNA and RNA synthesis Inhibition of topoisomerase II enzyme Generation of reactive oxygen species (ROS) Stabilization of microtubules	[49,51]
	Fluoropyrimidine + Taxane	Capecitabine + Docetaxel		
	Fluoropyrimidine + Epothilone	Capecitabine + Ixabepilone		
Adjuvant	Anthracycline + Taxane	Doxorubicin + Cyclophosphamide + Docetaxel		[49,52]

**Table 3:** Novel strategies for TNBC treatment.

Class of Agents	Examples of Therapy	Mechanism of Action	Refs.
PD-1 and PD-L1 inhibitors	Pembrolizumab + Paclitaxel Doxorubicin + Cyclophosphamide Pembrolizumab + Paclitaxel + Carboplatin Durvalumab + Nab-paclitaxel Atezolizumab + Nab-paclitaxel Atezolizumab + Nab-paclitaxel + Carboplatin	Reactivation of the anti-tumor immune response PD-1/PD-L1 complex formation inhibition	[55]
Platinum-based therapy	Carboplatin + Eribulin Gemcitabine + Carboplatin + Iniparib Carboplatin + Bevacizumab Cisplatin + Paclitaxel + Everolimus Paclitaxel + Carboplatin	Double-strand DNA break Apoptosis initiation	[56,57,58]
Cell cycle inhibitors	Trilaciclib, etoposide, abemaciclib, prexasertib	Activate the spindle assembly/mitotic checkpoint Prolonged mitotic arrest Cell death initiation	[59]
Angiogenesis inhibitors	Cisplatin + Bevacizumab Anlotinib, apatinib, afatinib, lenvatinib, erlotinib, famitinib, pyrotinib	Blocking new blood vessel formation Tumor growth inhibition VEGF signaling pathway disruption	[60]
PI3K/AKT/mTOR inhibitors	Rapamycin, ipatasertip, buparlisib, pictilisib, alpelisib	Cancer cells migration and invasion inhibition Apoptosis initiation	[61,62]
PARP inhibitors	Olaparib + Carboplatin + Paclitaxel Veliparib + Carboplatin Cisplatin + Rucaparib Veliparib, niraparib, talazoparib	Double-strand DNA break Cell death initiation Base excision repair Relax/condense chromatin bind nucleosom PARylate H1/H2B	[63]
EGFR inhibitors	Bintrafusp Alfa, dasatinib, geftinib, sorafenib, nimotuzumab, panitumumab, erlotinib, osimertinib	Cell death initiation Inhibition of cancer cell proliferation Blocking dimerization of receptors, auto-phosphorylation and downstream signaling Inducing receptor internalization, degradation and stable downregulation	[64]
Androgen receptor (AR) antagonists	Bicalutamide, enzalutamide, abiraterone, palbociclib	Decrease in cancer cell viability G1 phase arrest Apoptosis induction	[30,65]
Antibody drug conjugates	Sacituzumab govitecan, Ladiratuzumab vedotin, Trastuzumab deruxtecan	Cell growth and migration inhibition Binding to the topoisomerase in DNA replication inhibition S-phase-specific cell death initiation DNA damage	[66]

**Table 4:** Examples of miRNAs involved in TNBC and their specific targets.

miRNA	Regulation	Targets	Main Biological Mechanisms	Inflammasome Modulation	Refs.
Oncogenic miRNAs					
miR-135b	Up-regulated	APC, TGF-β	Promotes proliferation, invasion, migration and metastasis	Alters expression of inflammatory mediators (IL-1R1) Suppresses CASP1 expression following IL-1α stimulation	[86,87]
miR-21	Up-regulated	PTEN	Promotes TNBC cell proliferation Inhibits apoptosis	Modulates NLRP3 phosphorylation. Inhibits the assembly of NLRP3 inflammasomes	[110,111]
miR-301b	Up-regulated	CYLD	Promotes cell proliferation Resistance to apoptosis	Targets the TLR4/NF-kBsignaling pathway	[89,112]
miR-221	Up-regulated	FOSL1, MEK, ZEB2	Decreases the expression of epithelial-specific genes Increases the expression of mesenchymal-specific genes Suppresses oxidative	Suppression of NLRP3/ASC/CASP1 signaling pathway	[113,114]
miR-298	Up-regulated	P-gp	Drug efflux Promotes VEGF signaling Regulates NF-kBsignaling pathway	Regulates the secretion of pro-inflammatory cytokines	[115,116]
miR-449	Up-regulated	E2F1, E2F3, CDK2	Cell-cycle regulation	Regulation of NLRP3 inflammasome activation	[117,118]
miR-302b	Up-regulated	E2F1, ATM	Cell-cycle progression DNA damage repair	Inhibits IL-1β secretion and maturation Regulates the TLR/NF-kB signaling pathway	[119,120]
miR-663a	Up-regulated	HSPG2	Anti-apoptotic activity	Regulates the pro-inflammatory IL-1β expression	[90,91]
miR-638	Up-regulated	BRCA1	DNA damage repair	Involved in ATP synthesis-coupled electron transport	[92,121]
miR-137	Up-regulated	FSTL1	Wnt/β-catenin signaling Cellular stemness	Reduces the oxidative stress and inflammation via MAPK signaling pathway Modulates TLR4 expression levels	[121,122]
miR-140	Up-regulated	Wnt/β-catenin	Cellular stemness	Mediates inflammatory cytokines production Modulates TLR4 signaling pathway	[105,123]
miR-155	Up-regulated	CD44, CD90, ABCG2	Cellular stemness Drug efflux	Inhibition of TLR4/MyD88/NF-kB signaling	[14,124]
miR-210	Up-regulated	HIF1α	Promotes hypoxia	Modulates the necroptosis and pyroptosis in hypoxic conditions	[88,125]
miR-9	Up-regulated	ELAVL1, JAK1	Inhibition of EMT	Suppression of the NLRP3 inflammasome Inhibition of CASP1 expression and secretion of pro-inflammatory mediators	[11,126,127]
miR-181a	Up-regulated	ATG5, ATG22B	Autophagy Cellular stemness	Down-regulates the Bcl-2 expression Modulates the expression of IL-1	[11,128]
miR-373	Up-regulated	CD44	Invasion, intravasation, migration and metastasis	Modulates the activation of inflammasome via caspase-8	[84,85]
Tumor-Suppressor miRNAs					
miR-143-3p	Down-regulated	LIMK1	Inhibits cell growth, proliferation, migration and invasion	Regulates the MyD88/NF-kB signaling pathway Decreases the level of inflammatory mediators	[129,130]
miR-17-5p	Up or down regulated	ETV1, PDCD4, PTEN, DR4	Inhibits cell proliferation, invasion and apoptosis	Inhibits TXNIP/NLRP3 inflammasome pathway Inhibits pyroptosis	[131,132]
miR-217	Down-regulated	KLF5	Inhibits cell growth and migration	Modulates oxidative stress and pro-inflammatory cytokines release	[94,133]
miR-211-5p	Down-regulated	SETBP1	Cell proliferation, migration and metatasis	Inhibits pyroptosis Inhibits the expression of CASP1 and caspase-4	[134,135]
miR-185	Down-regulated	E2F6, DNMT1	Inhibits cell proliferation	Modulates the expression of NLRP family genes	[76,136]
miR-204-5p	Down-regulated	APL1S3	Promotes cancer cell aggressiveness	Decreases the CASP1 and ASC expression levels Inhibits the activation of NLRP3 inflammasome Suppresses the inflammatory responses	[137,138]
miR-128	Down-regulated	INSR, IRS1	Inhibits cell proliferation	Constrains the inflammasome assembly Modulates the pyroptotic cell death	[138,139]
miR-340	Down-regulated	SOX2, p16, p27	Cell cycle	Inhibits pyroptosis and inflammation Mediates the NEK7/NLRP3 signaling pathway	[95,96]
miR-384	Down-regulated	ACVR1	Inhibits cell proliferation and migration	Regulates inflammation	[109,140]
miR-200c	Down-regulated	XIAP	Inhibits cell proliferation promotes apoptosis	Decreases the NLRP3, CASP1, and GSDMD expression levels Inhibits NLRP3 inflammasome activation via NEK7 targeting Inhibits ATP and LPS-induced pyroptosis	[141,142,143]
miR-31	Down-regulated	BCL2, PKCε	Induces apoptosis Inhibits NF-kB signaling Modulates inflammation and oxidative stress	Inhibits caspase-1 activity Decreases NLRP3 and ASC expression levels Inhibits IL-1β and IL-18 releases	[97,98]
miR-200a	Down-regulated	PKCα, UBASH3B, XIAP	Suppresses proliferation, migration, invasion, and metastasis Promotes apoptosis	Regulates NOD2 expression	[116,144]
miR-141	Down-regulated	P27, CDK6, STAT5	Maintenance of epithelial phenotype	Modulates the expression of NLRP family genes	[76,145]
miR-27b-3p	Down-regulated	CBLB, GRB2	Regulation of PI3K/AKT and MAPK/ERK signaling	Attenuates NLRP3, CASP1, GSDMD, IL-1β and IL-18 and expression Anti-pyroptosic effects	[146,147]
miR-223	Down-regulated	HAX-1	Stimulates apoptosis Blocks the growth and the immunosuppressive ability of TNBC	Inhibits the activity of the NLRP3 inflammasome by binding the 3′-UTR of NLRP3 mRNA	[99,100]

Abbreviations: ATP-binding cassette super-family G member 2 (ABCG2), activin A receptor type I (ACVR1), autophagy related (ATG), ataxia telangiectasia mutated (ATM), casitas B-lineage lymphoma proto-oncogene-B (CBLB), cylindromatosis (CYLD), E2F Transcription Factor (E2F), embryonic lethal abnormal vision-like protein 1 (ELAVL1), Ets translocation variant 1 (ETV1), programmed cell death 4 (PDCD4), death receptor 4 (DR4), Fos-related antigen 1 (FOSL1), follistatin-related protein 1 (FSTL1), adaptor protein growth factor receptor-bound protein 2 (GRB2), hematopoietic lineage cell-specific protein-associated protein X-1 (HAX-1), hypoxia-inducible factor 1-alpha (HIF1α), heparan sulfate proteoglycan 2 (HSPG2), insulin receptor (INSR), insulin receptor substrate 1 (IRS1), Janus kinase 1 (JAK1), Krüppel-like factor 5 (KLF5), LIM kinase-1 (LIMK1), mitogen-activated protein kinase kinase (MEK), P-glycoprotein 1 (P-gp), protein kinase C (PKC), SET binding protein 1 (SETBP1), sex determining region Y-box 2 (SOX2), ubiquitin-associated and SH3 domain-containing protein B (UBASH3B), x-linked inhibitor of apoptosis protein (XIAP), zinc finger E-box-binding homeobox 2 (ZEB2).

## Data Availability

Not applicable.
